# A Case Report of Salmonella Typhi Osteomyelitis With Pathological Fracture in an Immunocompetent Adult

**DOI:** 10.7759/cureus.12211

**Published:** 2020-12-22

**Authors:** Venkataram V, Bhooma Subramanian, Manoharan Muthulingam

**Affiliations:** 1 Orthopedics and Traumatology, Mahatma Gandhi Medical College and Research Institute, Puducherry, IND; 2 Orthopedics, Mahatma Gandhi Medical College and Research Institute, Puducherry, IND; 3 Orthopedic Surgery, Mahatma Gandhi Medical College and Research Institute, Puducherry, IND

**Keywords:** salmonella typhi, chronic sclerosing osteomyelitis, tibia diaphysis, pathological fracture

## Abstract

Salmonella osteomyelitis is usually seen in patients with hemoglobinopathies and immunodeficient individuals. However, it is a rare clinical entity in an immunocompetent person with very few cases reported in clinical literature usually caused by non-typhoid Salmonella. Here, we report a case of *S. typhi* osteomyelitis of the right tibia in a 40-year-old immunocompetent lady. She developed a pathological fracture of the right tibia during the course of her treatment. It was then managed successfully by debridement and external fixation using a rail fixator. Salmonella osteomyelitis does not present with unique clinical or radiological signs. High index of suspicion with appropriate testing will help in determining the causative agent correctly and thus aid in its successful management.

## Introduction

Salmonella are flagellated, rod-shaped, gram-negative facultative anerobes. Salmonella genus is split into two species: *Salmonella enterica* and *Salmonella bongori*. *S. enterica* is further divided into six subspecies. Around 2500 serotypes have been described based on differences in H (flaggellar) and O (surface) antigens. Approximately 99% of all infections are caused by *S. enterica* [1]. They are broadly classified into three types: non-invasive non-typhoidal salmonellosis, invasive non-typhoidal salmonellosis, and typhoid fever.

Regardless of the primary illness, Salmonella species as a causative agent of osteomyelitis is mostly seen in individuals with hemoglobinopathies and immunosuppressive disorders. It is very rarely seen in immunocompetent adults [2-5]. The clinical and radiological presentation is also indistinguishable from osteomyelitis caused by other common microorganisms. Here, we report an unusual case of Salmonella osteomyelitis of the tibia complicated by pathological fracture in a middle-aged woman without sickle cell disease or any other predisposing factors contributing to immunosuppression.

## Case presentation

**Figure 1:**
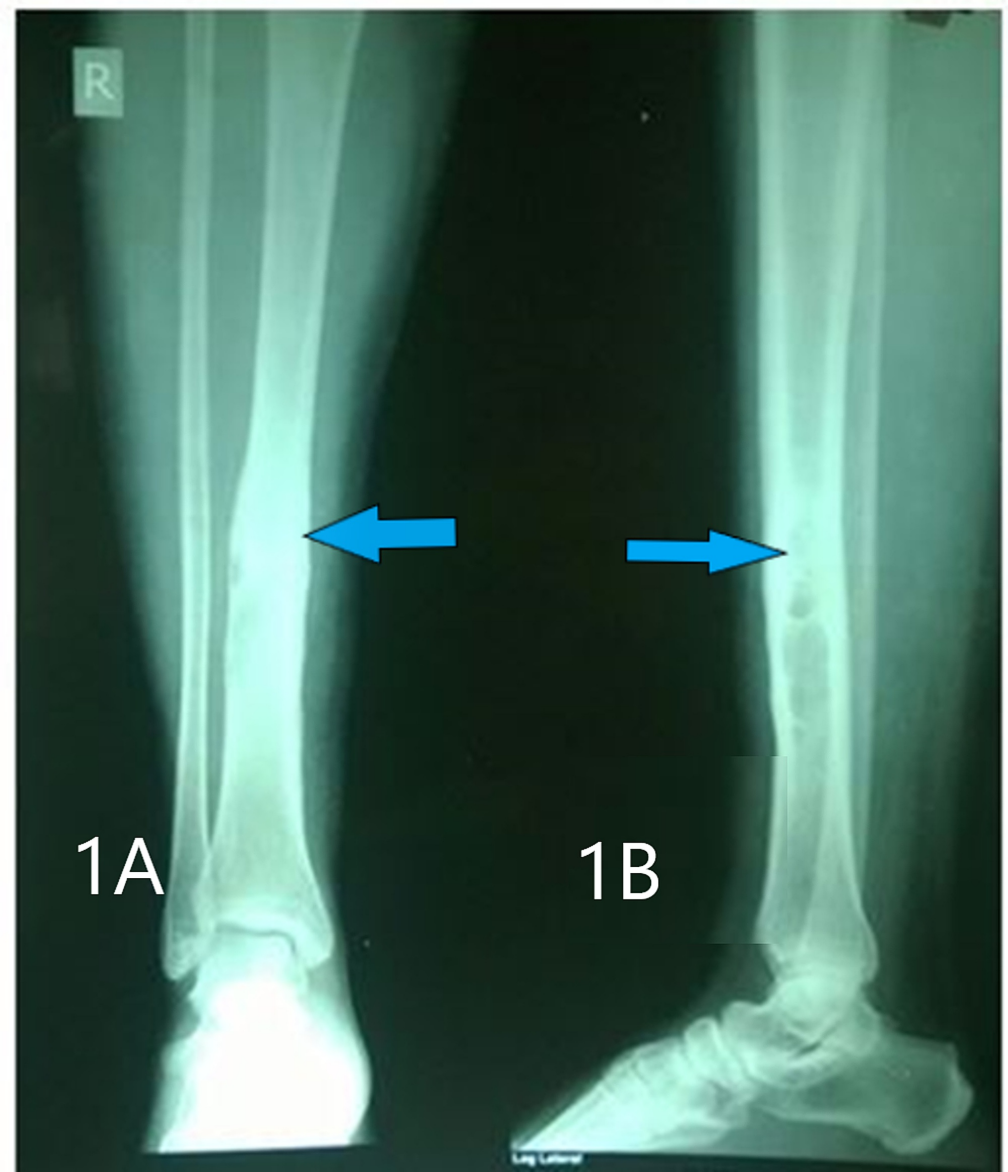
Pre-operative X-ray showing changes suggestive of osteomyelitis. Figures 1A, 1B show the anteroposterior and lateral views of the right tibia showing periosteal thickening, loss of corticomedullary differentiation, and lytic changes.

A 40-year-old female patient was admitted to our hospital with complaints of fever for a duration of three days, pain and swelling in right lower limb for one week. On examination, a swelling of size 5 cm × 3 cm was palpated over the distal half of the right leg. The skin was stretched and shiny. It was warm and tender to touch. Thickening and bony irregularity in distal part of tibia was felt. Routine blood investigations showed total leukocyte count of 12,300 cells/mm^3^, erythrocyte sedimentation rate (ESR) was found to be 90 mm at the end of one hour, and qualitative C-reactive protein test yielded a positive result. Based on the clinical, radiological, and hematological findings, infection was suspected. Hence blood culture was done, but it yielded no growth. Radiographic findings showed diffuse thickening of cortex in the distal tibia with loss of cortico-medullary differentiation and patchy areas of lysis as shown in Figure 1.

Provisional diagnosis of chronic osteomyelitis with acute exacerbation was made. The patient was taken up for surgical debridement. Cortical window of size 1.5 cm × 1.5 cm was made in the distal tibia, and frank pus was evacuated. Thorough reaming and irrigation were done. Post-operatively patient was placed on an above-knee slab and was advised strict nonweight-bearing.

Pus culture yielded *S. typhi*, which was sensitive to the commonly used antibiotics. Peripheral blood smear showed neutrophilic leukocytosis and mild eosinophilia. Blood, urine, and feces culture had no growth.

**Figure 2:**
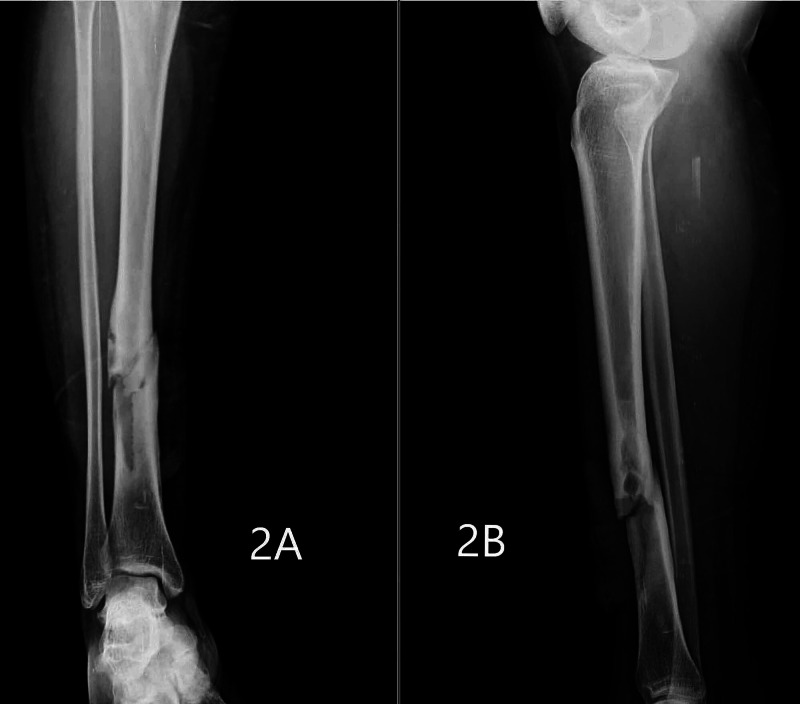
Pathological fracture of the right tibia Figures 2A, 2B show pathological fracture of right tibia in anteroposterior and lateral views, respectively.

The patient showed good clinical response to culture-specific antibiotic therapy. Inj. ceftriaxone 2 g daily was given for two weeks and then converted to oral ciprofloxacin 500 mg twice daily. One month following surgical debridement, the patient sustained a pathological fracture following a trivial fall while going to the bathroom as shown in Figure 2.

**Figure 3:**
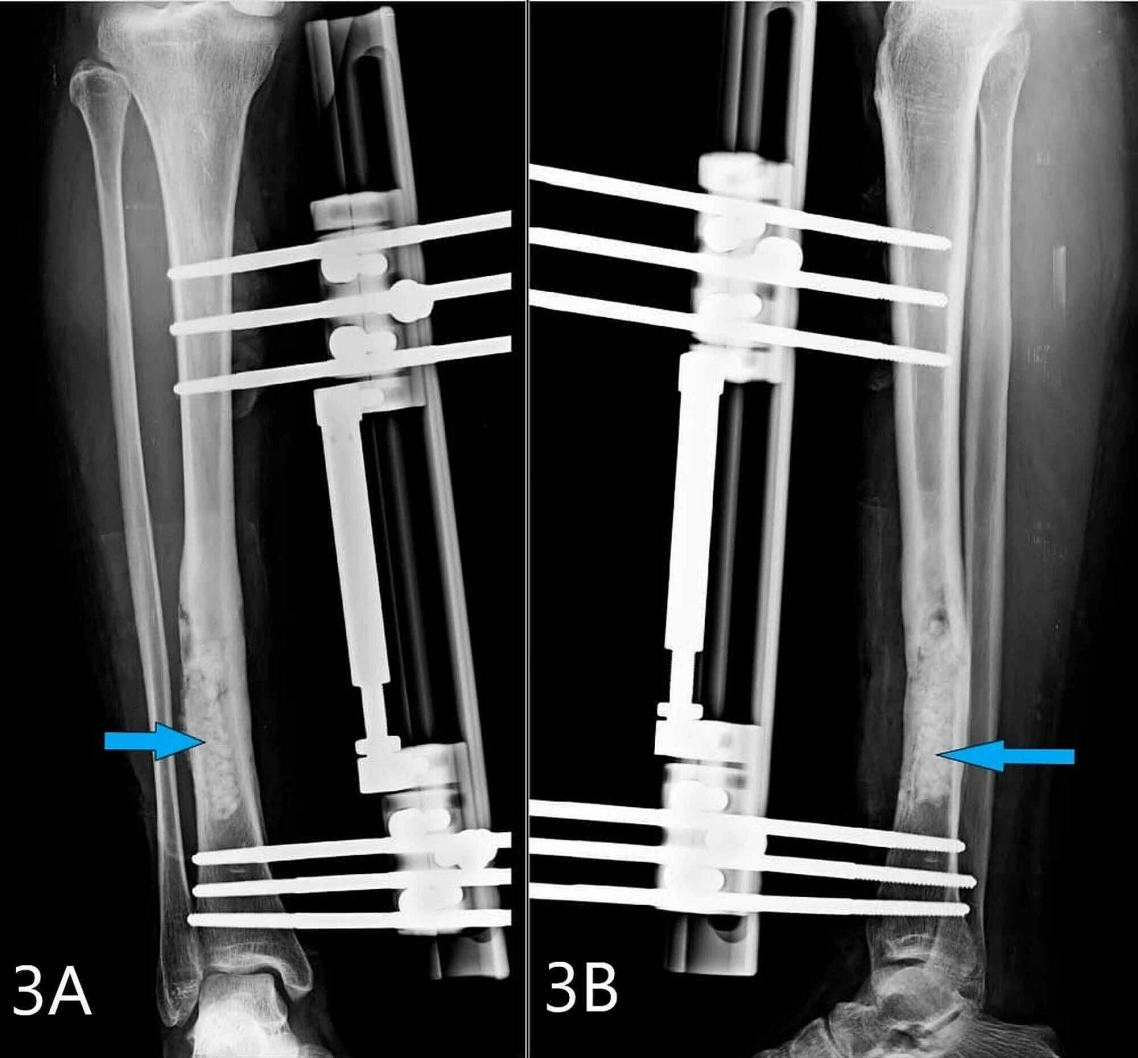
Post-operative X-ray taken after surgery Figures 3A, 3B show post-operative X-ray showing the fixation with LRS and arrow pointing at the bio-composite antibiotic beads. LRS, Limb reconstruction system.

She then underwent debridement to remove unhealthy granulation tissue, bio-composite antibiotic beads placement, and external fixation using limb reconstruction system (LRS) fixator. Figure 3 shows the post-operative X-ray taken after surgery. Intravenous ceftriaxone was stopped after day two, and the patient was continued on oral ciprofloxacin for two weeks. Post-operatively patient was kept on nonweight-bearing for six weeks and then started on partial weight-bearing after check X-ray showed signs of fracture union.

**Figure 4:**
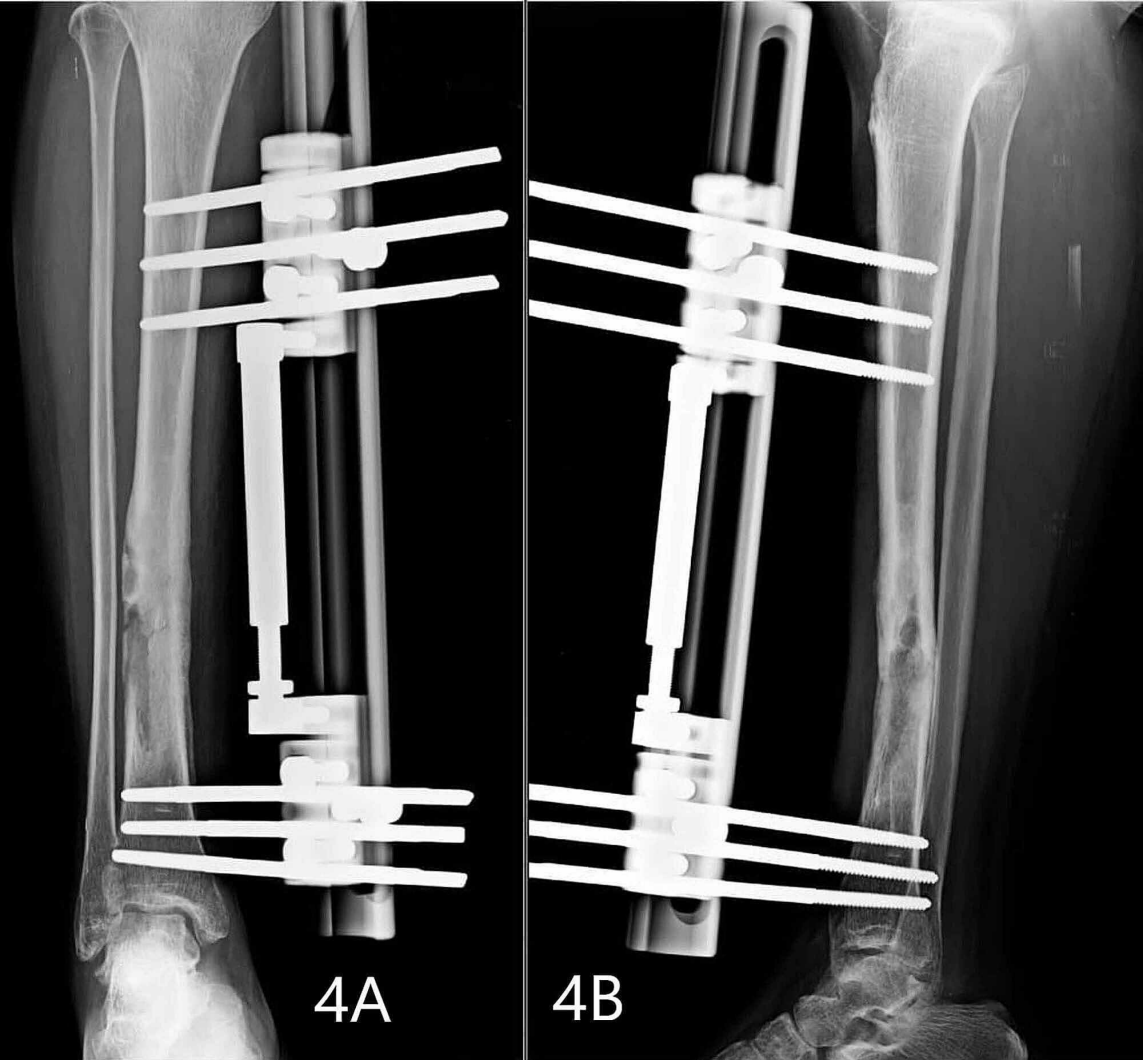
Fracture showing good union at the end of three months post-op. Figures 4A, 4B show good fracture union in anteroposterior and lateral views, respectively. The beads have already been fully resorbed.

Fracture united in three months with no wound complications. The fixator was removed after three months, and the patient was put on below-knee walking cast to protect the bone. Figure 4 shows the uniting tibia with the LRS in situ.

**Figure 5:**
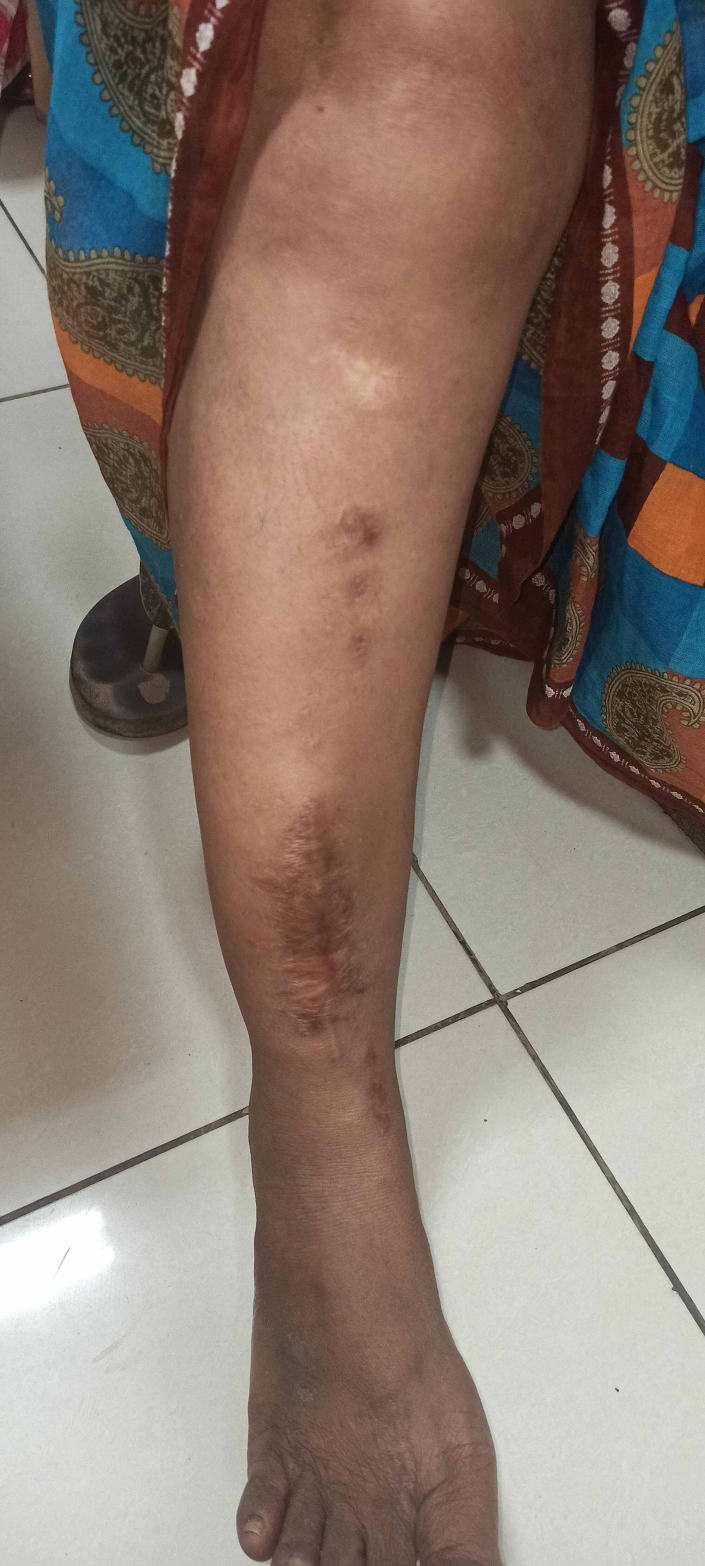
Clinical picture of the right leg at latest follow-up showing healed surgical scar and pin tract scars.

**Figure 6:**
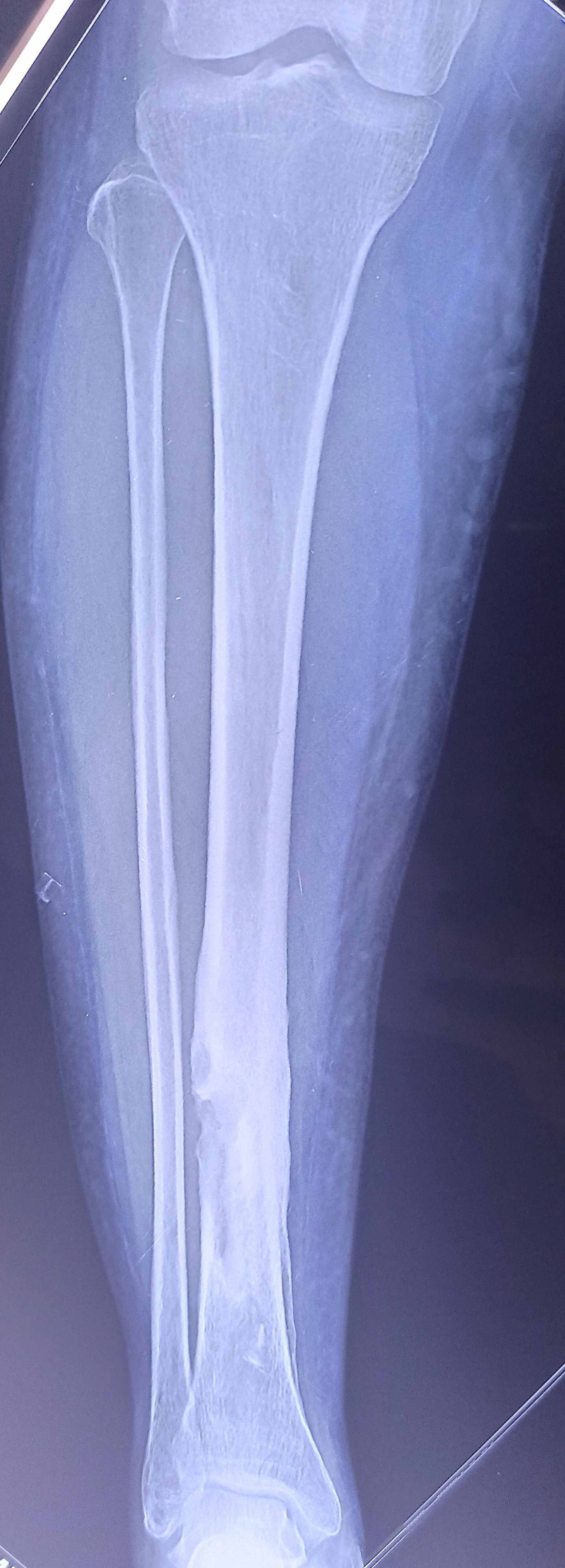
X-ray taken at follow-up showing healed pathological fracture of the right tibia.

Patient is still under follow-up. She did not have any recurrence and has returned to her normal day-to-day activities with full functional capabilities. Figures 5, 6 show the clinical picture and X-ray of her right leg that was taken at the latest follow-up two years since primary surgery.

## Discussion

Salmonella-related infections can be broadly classified into two types based on the history and clinical presentation. Non-typhoid Salmonella infections (NTS) and enteric fever: The former predominantly presents as gastroenteritis with fever, bloody diarrhea, and abdominal cramps. The latter is the classical typhoid illness presenting with fever, malaise, headache, and lethargy. However, both can be involved in bacteremia and present with focal infections such as osteomyelitis, meningitis, and urinary tract infections [6].

Salmonella osteomyelitis is very rare in normal individuals constituting 0.8% of all Salmonella infections and only 0.45% of all osteomyelitis cases [2]. The infection is usually associated with hemoglobinopathies such as sickle cell disease, malignancies, and immune deficiencies. The three most common Salmonella serovars causing osteomyelitis are *Salmonella typhimurium*, *S. typhi*, and *Salmonella enteritidis* [7]. Most common mode of spread of infection is hematogenous, and the most common sites involved are the diaphysis of long bones.

Radiographic findings were consistent with osteomyelitis with periosteal thickening, loss of corticomedullary differentiation, and some lytic changes. It is important to note that none of these findings are specific for Salmonella osteomyelitis. The growth of gram-negative rods along with the characteristic morphologic findings confirmed the etiology as Salmonella. One of the supporting investigations in our case was Widal test positivity. Although blood culture was negative for Salmonella sp. in our case, positive blood culture results have been reported in up to 30% of patients with Salmonella osteomyelitis [8].

Very few studies report osteomyelitis caused by Salmonella organisms [3,4,9,10]. Even among them, non-typhoid Salmonella was the most common causative organism usually preceded by a history of gastroenteritis.

Osteomyelitis in long bones is a consequence of hematogenous spread. The general consensus is that appropriate intravenous antibiotic therapy with or without surgery usually gives a good clinical outcome [3,10]. Recurrence is unusual in the absence of hemoglobinopathies [11].

Pathological fractures following osteomyelitis have been reported a few times in scientific literature [12,13]. The combination of bone destruction by the infection along with defects created by surgery makes it especially vulnerable for pathological fractures to occur. However, they are still not as common as what one would expect. The principles of management in such a scenario are the same, irrespective of the etiology, control of infection, temporary stabilization, and definitive fixation after infection control [14].

We aimed to achieve infection control thorough debridement and placement of bio-absorbable antibiotic beads impregnated with the culture-specific antibiotics. Stabilization and definitive fixation were achieved simultaneously by the usage of rail fixator. Early identification of the causative organism and usage of appropriate antibiotics played an important role in the successful management of this condition and its complication, which is otherwise known for being notoriously difficult to manage [13,14].

## Conclusions

*S. typhi* osteomyelitis is clinically and radiologically indistinguishable from osteomyelitis caused by other organisms. Appropriate duration of culture-sensitive antibiotics combined with operative intervention on a case-to-case basis is the ideal management strategy. Clinicians need to be aware of the risk of complications such as pathological fracture especially after surgical decompression. Ensuring infection control by adequate debridement, skeletal stabilization by an external fixator (rail fixator or Ilizarov ring fixator, GPC Medical Ltd., India), and avoiding internal fixation will ensure a good clinical outcome even in the event of such a complication.

Salmonella osteomyelitis is getting reported in immunocompetent patients in recent times. Hence it is prudent to consider Salmonella as a possible etiological agent in adult diaphyseal osteomyelitis and prepare a workup accordingly. As our case report shows early identification of the causative organism and administration of appropriate antibiotics ensures a successful outcome.
